# Early Identification of Low Scorers: The Role of Formative Assessments and Summative Assessments

**DOI:** 10.7759/cureus.76118

**Published:** 2024-12-21

**Authors:** Himel Mondal, Devendra Nath Tiu, Ayesha Juhi, Shaikat Mondal

**Affiliations:** 1 Physiology, All India Institute of Medical Sciences, Deoghar, IND; 2 Physiology, Sheikh Bhikhari Medical College, Hazaribagh, IND; 3 Physiology, Raiganj Government Medical College and Hospital, Raiganj, IND

**Keywords:** assessment, comprehension, educational measurement, formative, low achievers, low score in examination, medical students, prediction, summative, university examination

## Abstract

Background

Identifying students who are low scorers is essential for providing timely interventions and support. Traditional methods of identifying such students often rely on summative assessments, such as end-of-term exams. However, formative assessments offer valuable insights into students' understanding, strengths, and weaknesses. This study aimed to compare the predictive capabilities of formative and summative assessments in forecasting final university examination scores in physiology among first-year medical students. By employing a regression model, we aimed to develop a reliable predictor of final scores based on formative and summative assessments. Such a model would enable the early identification of students at risk of obtaining low scores in university examinations.

Methods

A cohort of 125 first-year medical students was evaluated. Scores from three formative assessments, three summative assessments, and the final university examination in physiology were collected. The formative assessments consisted of multiple-choice questions (MCQs), while the summative assessments and university examinations included a combination of MCQs and essay-type questions. Correlation and regression analyses were performed to determine the predictive power of these assessments.

Results

Data from 82 students were analyzed. The highest mean score was observed in the final university examination (63.28 ± 4.63), followed by the third summative assessment (62.45 ± 8.2). Significant differences were found among the scores across the various assessments (F (4.587, 371.5) = 61.14, p-value < 0.0001). Formative assessments and summative assessment scores can predict the university examination score (R = 0.764, R^2^ = 0.583, p-value < 0.0001). However, individually, only the second summative (t = 2.87, p-value = 0.005, 95% confidence interval = 0.038-0.21) and the third summative (t = 3.25, p-value = 0.002, 95% confidence interval = 0.086-0.359) significantly contribute to the model of prediction.

Conclusion

Students' performance in formative and summative assessments can predict success in university exams, with the second and third summative assessments being significant. These assessments, conducted later in the course, are more effective in identifying students likely to score lower in final exams compared to formative assessments.

## Introduction

In medical education, identifying students at risk of underperforming is essential for providing timely interventions and support [1]. Traditional methods for identifying these students often rely heavily on summative assessments, such as end-of-term exams [2]. While summative assessments offer a comprehensive evaluation of students' knowledge, they are conducted infrequently and typically at the end of a learning period. Consequently, they provide little opportunity for early intervention. However, early identification of the underachievers is important as they may have multiple issues such as health concerns, difficulties with learning the English language, difficulties with online learning, and learning obstacles [3].

Formative assessments, on the other hand, are designed to monitor student learning throughout the course and provide continuous feedback. These assessments include a variety of formats, such as quizzes, assignments, and in-class activities, and are aimed at identifying students' comprehension, strengths, and weaknesses on an ongoing basis [4]. Research has shown that formative assessments can enhance learning and improve student outcomes by allowing for real-time adjustments to teaching strategies and study habits [5].

Heggarty et al. reported that objective formative assessments can predict success in the fellowship exams offered by the Royal Australian College of General Practitioners. Hence, they suggested that their formative assessment can be used as a tool to predict academic achievement and to identify trainees who may struggle early on [6]. In support of that, Zhang et al. also found that formative assessments can successfully predict summative assessments among students at Palmer College of Chiropractic [7].

Despite the acknowledged benefits of formative assessments, there is a literature gap regarding the evidence of their predictive capability of the final examination performance of Indian medical students. With this background, this study aimed to fill this gap by exploring the relationship between formative assessment and summative assessment scores and final university examination scores in physiology among first-year medical students. By understanding this relationship, educators can potentially identify the tests that can be used to identify low-scoring students for targeted interventions that could improve their academic performance. By employing a regression model, we aimed to develop a reliable predictor of final scores based on formative and summative assessments. Such a model would enable the early identification of students at risk of obtaining low scores in university examinations. Specifically, if a student performs poorly in formative assessments, assuming these scores significantly contribute to the model, they could be flagged as potential low performers in the final university evaluation.

## Materials and methods

Study design and setting

In this study, we took a cohort of students for one year, starting in August 2022 and ending in July 2023. The data were collected for the academic year from first-year undergraduate medical students studying in an institution of national importance. This year, students learned three basic science subjects: anatomy, physiology, and biochemistry. This study was conducted after approval from the institutional ethics committee (approval number 2023-212-IND-03).

Study participants

The study participants for this study consist of 125 students enrolled in the first year of the Bachelor of Medicine, Bachelor of Surgery (MBBS) program. They were recruited from the beginning of the session, where their examination scores were collected from the departmental and university registers.

Assessment tools

**Figure 1:**
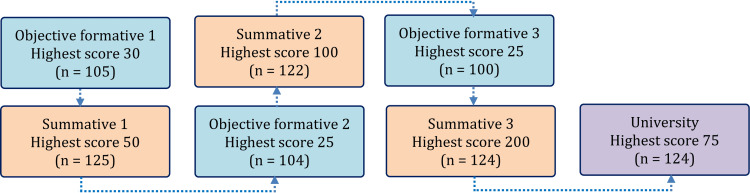
Assessments used in the study with the number of students in brackets n: number

This study utilized three types of assessment tools: formative assessments, summative assessments, and the final university examination. The formative assessments consisted of three structured, objective tests, each comprising multiple-choice questions (MCQs) designed to evaluate students' understanding of fundamental physiology concepts. These objective-type assessments were specifically chosen to eliminate potential assessor bias. The summative assessments included three evaluations, each featuring a combination of MCQs and essay-type questions, aimed at assessing both comprehensive knowledge and application skills in physiology. Similarly, the final university examination incorporated a mix of MCQs and essay-type questions, closely mirroring the format of the summative assessments. The structure of the assessments used in this study is depicted in a flowchart (Figure 1).

Data collection

Scores from the three formative assessments, three summative assessments, and the final university examination were collected for each student. The MCQ formative assessments were of 30 marks, 25 marks, and 25 marks. The semester examinations had theory 50 marks, 100 marks, and 200 marks. The final professional examination had 75 marks for theory. Some of the students were absent from different examinations. Hence, incomplete entries were identified and removed from the final analysis. All assessments were scored out of a total of 100 to make it uniform for comparison. The scores of the formative and summative assessments were collected from the departmental registry. The university examination scores were obtained with special permission from the university database.

Data analysis

Pearson correlation coefficients were calculated to assess the relationship between scores from each formative assessment, summative assessment, and the final university examination. Regression analysis was performed to determine the predictive power of the formative and summative assessment scores on the final university examination scores. The regression model included scores from the three formative assessments and three summative assessments as independent variables, with the final examination score as the dependent variable. There were six examinations, with one university examination. Many of the students were present in one examination and not in another examination, especially in formative assessments. We only included the data of students who completed all the examinations. Hence, the number of students was 82. The upper 27% is considered a high scorer, the lower 27% is considered a low scorer, and others are average scorers, and their scores were compared by one-way analysis of variance (ANOVA). Furthermore, a one-way repeated-measures ANOVA was used to compare the scores of formative assessments, summative assessments, and university scores. Tukey’s post-hoc test was employed to identify significant differences between the groups. The statistical tests were conducted in GraphPad Prism 9.5.0 (GraphPad Software Inc., San Diego, CA), and a p-value <0.05 was considered statistically significant.

## Results

The data of 82 students were analyzed. Students obtained the highest score (63.28 ± 4.63) in the university examination, followed by the third summative assessment (62.45 ± 8.2). When students were divided into three categories according to their scores in the final university examinations, the scores showed a similar pattern except for high scorers (Table 1).

**Table 1:** Mean score of assessment among the overall students and high, average, and low scorering students * F-value, R^2^, and p-value are used for the analysis of variance (ANOVA) of scores among low, average, and high scorers. The upper 27% of scorers were considered high scorers, the lower 27% of scorers were considered low scorers, and others were average scorers. SD: standard deviation, n: number

Examination	Overall (n = 82)	Low scorers (n = 22)	Average scorers (n = 38)	High scorers (n = 22)	F-value, R^2^, p-value*
	Mean ± SD				
Formative 1	43.63 ± 12.92	28.3 ± 4.02	42.6 ± 4.76	60.73 ± 5.79	244.5, 0.86, <0.0001
Formative 2	56.1 ± 14.38	37.55 ± 11.94	58.58 ± 5.07	70.36 ± 4.96	108.8, 0.73, <0.0001
Formative 3	60.59 ± 11.08	46.91 ± 7.87	61 ± 3.59	73.55 ± 4.09	144.9, 0.79, <0.0001
Summative 1	52.9 ± 10.39	40 ± 6.82	53.53 ± 2.65	64.73 ± 6	133.9, 0.77, <0.0001
Summative 2	50.66 ± 12.62	34.34 ± 7.87	51.79 ± 4.51	65.02 ± 4.36	168.2, 0.81, <0.0001
Summative 3	62.45 ± 8.2	51.95 ± 5.77	63.17 ± 2.54	71.68 ± 2.3	151.6, 0.79, <0.0001
University	63.28 ± 4.63	57.52 ± 2.19	63.33 ± 1.69	68.97 ± 2.09	192.7, 0.83, <0.0001

There was the highest level of positive correlation between the score of the university examination and the summative assessment conducted just before the university examination. Overall, all the formative assessments had lower coefficients than summative assessments. The correlation of university examinations with formative assessments and summative assessments is shown in Table 2.

**Table 2:** Correlation of university examination theory scores with formative and summative assessments r: Pearson's correlation coefficient, CI: confidence interval, R^2^: coefficient of determination

Pearson's correlation parameters	Formative 1	Formative 2	Formative 3	Summative 1	Summative 2	Summative 3
r	0.43	0.42	0.42	0.53	0.7	0.71
95% CI	0.24 to 0.59	0.22 to 0.58	0.23 to 0.59	0.35 to 0.67	0.56 to 0.79	0.58 to 0.8
R^2^	0.186	0.175	0.178	0.28	0.485	0.503
p-value	<0.0001	<0.0001	<0.0001	<0.0001	<0.0001	<0.0001

**Figure 2:**
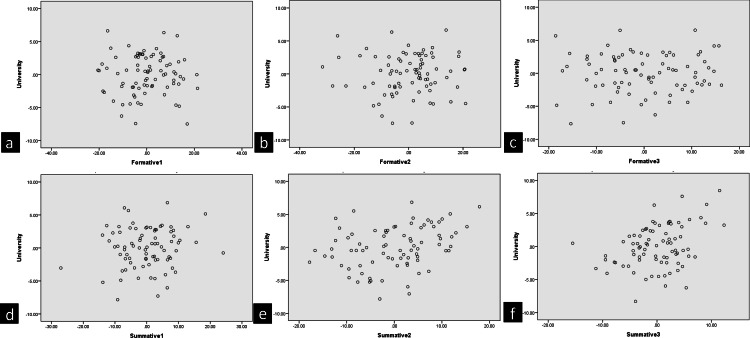
Scatterplot between university examination score versus formative assessment 1 (a), formative assessment 2 (b), formative assessment 3 (c), summative assessment 1 (d), summative assessment 2 (e), and summative assessment 3 (f)

Scores of formative assessment and summative assessment can predict the university examination score (R = 0.76, R^2^ = 0.58, p-value < 0.0001). However, individually, only summative 2 (t = 2.87, p-value = 0.005, 95% CI = 0.04-0.21) and summative 3 (t = 3.25, p-value = 0.002, 95% CI = 0.09-0.36) significantly contribute to the model of prediction. None of the formative assessments were significantly contributing to the prediction model. The scatterplot between scores of university examinations and formative (Figures 2a, 2b, 2c) and summative (Figures 2d, 2e, 2f) is shown in the Appendix (Figure 2).

## Discussion

The primary finding of this study is that students achieved their highest scores in the university examination, closely followed by the third summative assessment. The significant differences observed among the various assessments highlight the variability in student performance across different types of evaluations. The predictive model developed in this study demonstrated that the combined scores from formative and summative assessments can effectively predict university examination scores. However, individual analysis revealed that only the second and third summative assessments significantly contributed to this model. This finding is consistent with the notion that summative assessments, designed to evaluate comprehensive knowledge and skills acquired over time, provide a more accurate reflection of a student's readiness for final exams than formative assessments. A study conducted at the University of Ottawa, Canada, revealed a moderately positive correlation between formative MCQs and final examinations and a relatively strong correlation between midterm and final examinations [8]. Hence, supporting our study, this study also found that examinations conducted at the end of the year can better predict the final performance. Furthermore, a study conducted at Massachusetts College of Pharmacy and Health Sciences, USA, suggested that the formative assessment score can predict the summative score of the Physician Assistant National Certifying Examination [9].

When we tested the correlation between the final university examination and formative and summative assessment, we found that all had statistically significant correlations. However, considering the small sample size, the correlation coefficients were quantitatively weak. A study from the University of Ribeirão Preto found that, in problem-based learning, the score in formative assessment has a positive correlation with summative objective structured clinical evaluation and progress testing [10]. In contrast, a study conducted at Sunderland Royal Hospital, UK, reported that, although there are intra-test correlations in formative assessments or summative assessments, there is no significant correlation between overall grades for the formative and summative assessments [11]. This discordant funding may be due to multiple factors such as test type, course syllabus, institutional variation, geographical and cultural variation, differences in teaching-learning methods, and many more. Hence, this topic needs further exploration with a uniform teaching-learning environment.

In our study, we found that almost 20% of the students were absent in the formative assessment sessions. A study by Kühbeck et al. done on a cohort of students enrolled in pharmacology courses at the Technical University of Munich, Germany, found that, when students participate in multiple formative assessments, their frequency of participation is not correlated with the summative assessment score [12]. In contrast, Winston et al. reported that, when the low scorers are offered discussion sessions, a single session is not effective, but attending more than three follow-up small group sessions significantly improves pass rates two semesters later [13]. From the above two studies, it is evident that occasional participation may not yield immediate benefits. Hence, a consistent and structured engagement with formative activities with timely feedback is crucial for improving outcomes over time [14,15].

This study provides novel insights into the predictive power of different types of assessments on university examinations from an Indian medical institution. However, the study's limitation lies in its sample size and the focus on a single subject within one university, which may not generalize across diverse educational contexts or disciplines. Further research involving a larger, more varied sample and multiple subjects is necessary to validate these findings and extend their applicability.

## Conclusions

Students' performance in formative and summative assessments is a significant predictor of their success in university examinations. However, the scores of the second and third summative assessments, which were conducted late in the one-year course, were significantly predicting the final examination score. This suggests that, while all assessments contribute to learning and overall predict the final examination score, students who would score low in the final university examination can be identified from summative assessments that are conducted mid- and end-year. However, this result is for a particular subject in a university in India. Further studies are needed to explore the capability of the formative and summative assessments in predicting final examination scores aimed at identifying low-scoring students in different settings and different subjects.
